# Vaginal *Lactobacillus iners* Abundance Predicts Response to Local Estrogen Therapy in Postmenopausal Women with Genitourinary Syndrome of Menopause

**DOI:** 10.3390/microorganisms14081760

**Published:** 2026-08-10

**Authors:** Xu Han, Baiyun Sun, Yan Zhu, Yanan Liu

**Affiliations:** Department of Obstetrics and Gynecology, The Second Affiliated Hospital of Soochow University, Suzhou 215004, China; 15895530066@163.com (X.H.);

**Keywords:** genitourinary syndrome of menopause, local estrogen therapy, *Lactobacillus iners*, vaginal microbiota, treatment response

## Abstract

Genitourinary syndrome of menopause (GSM) is a common chronic condition in postmenopausal women, yet biomarkers predicting response to local estrogen therapy remain insufficiently defined. In this prospective study, 216 postmenopausal women with GSM received local estradiol vaginal tablet therapy for 12 weeks. Baseline assessments included symptom evaluation, vaginal pH, vaginal maturation index (VMI), and quantitative real-time PCR measurement of six target microorganisms. Treatment response was defined as an improvement of at least 2 points in the most bothersome symptom (MBS) score. After 12 weeks, MBS score, vaginal pH, and VMI improved significantly, with increased abundance of *L. crispatus*, *L. jensenii*, and *L. gasseri* and decreased abundance of *L. iners*, *Gardnerella*, and *Candida*. Overall, 128 of 216 women responded (59.3%; 95% CI, 52.6–65.6%). Higher baseline *L. iners* abundance was independently associated with a lower probability of response (adjusted RR per log10-unit increase, 0.82; 95% CI, 0.70–0.95), with the highest tertile showing a lower response likelihood than the lowest (RR, 0.71; 95% CI, 0.54–0.93). These findings suggest that *L. iners* may be a promising candidate microbial marker for pretreatment risk stratification, although external validation is needed before clinical application.

## 1. Introduction

Genitourinary syndrome of menopause (GSM) is a common and persistent chronic health condition in postmenopausal women, primarily caused by estrogen deficiency. It may involve multiple anatomic sites, including the vulva, vagina, urethra, and bladder, and commonly manifests as vaginal dryness, burning, irritation, dyspareunia, urinary frequency, urgency, and recurrent urinary discomfort [1]. GSM is highly prevalent and often has a sustained impact on quality of life, sexual function, and overall well-being. The 2020 NAMS/ISSWSH position statement reported that GSM affects approximately 27% to 84% of postmenopausal women, underscoring its clinical importance in long-term postmenopausal care [2].

Local estrogen therapy is one of the established treatments for improving GSM-related symptoms and local vaginal signs. It can effectively relieve vaginal symptoms, lower vaginal pH, and promote vaginal epithelial maturation [2]. However, clinical responses to local estrogen therapy are not uniform, and substantial heterogeneity in treatment response is observed in practice [3]. Previous studies have largely focused on the overall efficacy of local estrogen therapy, whereas evidence remains limited regarding which patients are most likely to benefit and which baseline biological features may predict treatment response. Therefore, identifying pretreatment markers of response heterogeneity has clear clinical relevance.

With declining estrogen levels after menopause, vaginal glycogen decreases, *Lactobacillus* dominance becomes less prominent, vaginal pH increases, and the vaginal microbial community tends to become more diverse and unstable [4,5]. Local estrogen therapy may partially reverse these changes by improving the vaginal microenvironment and promoting enrichment of *Lactobacillus*-related taxa [6,7]. However, not all *Lactobacillus* species have equivalent biological significance. Whereas *L*. *crispatus* is generally associated with a more stable and protective vaginal ecosystem, *L*. *iners* is frequently observed in transitional states between vaginal health and dysbiosis and may not confer the same ecological stability [8,9]. Its role in postmenopausal women with GSM, particularly in relation to treatment response, remains unclear. Therefore, this study aimed to evaluate the association between baseline vaginal microbial abundance and response to local estrogen therapy, with a particular focus on the potential relevance of *L. iners*.

## 2. Materials and Methods

### 2.1. Study Population

**Figure 1:**
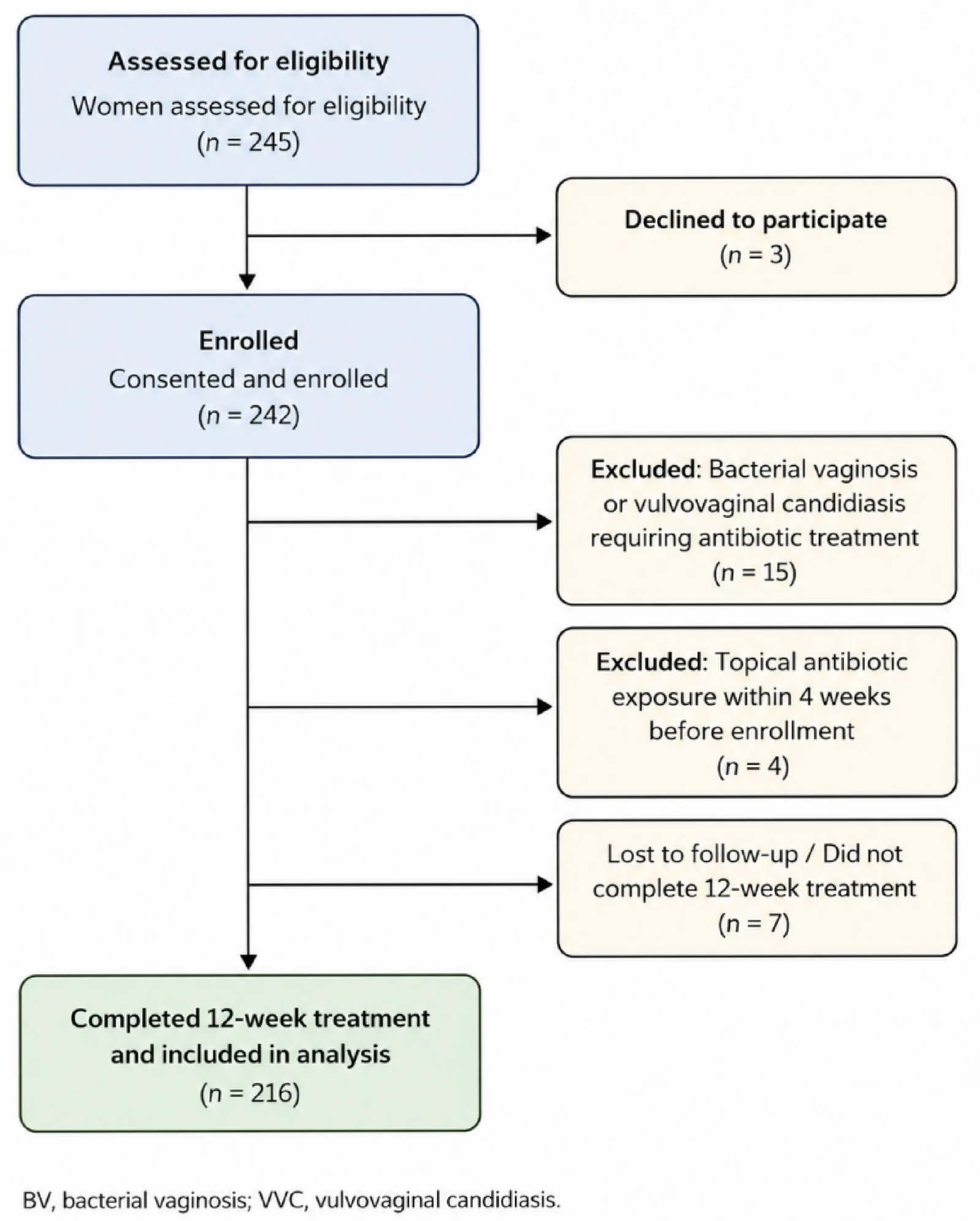
Flow diagram of participant enrollment.

This study enrolled patients with GSM who attended the gynecologic endocrinology outpatient clinic of The Second Affiliated Hospital of Soochow University. Participants were enrolled between June and December 2025, and all 12-week follow-up assessments were completed after enrollment of the final participant. The inclusion criteria were as follows: (1) naturally postmenopausal women aged >45 years who reported amenorrhea for at least 12 consecutive months; (2) presence of at least one subjective symptom, including vaginal dryness, dyspareunia, burning, irritation, or itching; (3) at least one objective finding detected by vaginal swab assessment, including vaginal pH > 5.0, reduced superficial cells on the vaginal maturation index (VMI); and (4) willingness to receive a standardized local estrogen regimen. The exclusion criteria were as follows: (1) bacterial vaginosis or vulvovaginal candidiasis confirmed by routine vaginal discharge examination and requiring antibiotic treatment; (2) refusal of vaginal swab sampling or follow-up; (3) reported oral or local vaginal antibiotic use within 4 weeks before enrollment; (4) sexual intercourse, vaginal douching, or cervical/vaginal procedures within 3 days before sampling; (5) confirmed vaginal human papillomavirus infection; and (6) contraindications to local estrogen therapy. All participants provided written informed consent, and the study protocol was reviewed and approved by the Ethics Committee of The Second Affiliated Hospital of Soochow University (approval No: JD-HG-2025-058). We recruited 245 potential participants. Ultimately, 216 patients who met the inclusion and exclusion criteria signed the informed consent form and were enrolled in the study cohort (Figure 1).

### 2.2. Baseline Assessment

At baseline, all participants completed a standardized questionnaire. The questionnaire collected information on age, height, weight, years since menopause, sexual activity, smoking status, history of chronic diseases (diabetes and hypertension), and history of recurrent vaginitis. The most bothersome symptom (MBS) was also assessed. Symptoms included common vulvovaginal manifestations of GSM, namely vaginal dryness, dyspareunia, burning, itching, and irritation. Each participant identified the symptom that had the greatest impact on daily life or caused the greatest subjective discomfort, which was defined as the MBS. MBS severity was assessed using a 4-point scale: 0 indicated no symptom, 1 mild, 2 moderate, and 3 severe.

Vaginal swabs were collected for assessment of the VMI, screening for bacterial vaginosis and vulvovaginal candidiasis, pH measurement, and laboratory quantification of bacterial load. During sampling, participants were placed in the lithotomy position, and the vagina was exposed using a speculum. Three sterile cotton swabs were gently rotated against the lateral wall of the upper one-third of the vagina until fully saturated with secretions. For VMI assessment, samples were evenly smeared, fixed, and stained using the Papanicolaou method. Cytological interpretation was performed under light microscopy by uniformly trained laboratory personnel. Vaginal epithelial cells were classified by morphology as superficial cells, intermediate cells, and parabasal cells. At least 100 epithelial cells were counted per specimen, and the percentages of the three cell types were calculated. VMI was expressed as the relative proportions of superficial, intermediate, and parabasal cells. Because the proportion of superficial cells is a sensitive indicator of local estrogenic effect and vaginal epithelial maturation, this study used the proportion of superficial cells as the primary indicator of VMI change.

### 2.3. Quantification of Microbial Abundance

Immediately after collection, specimens were placed in sterile preservation tubes and transported to the laboratory under cold-chain conditions. Microbial nucleic acids were extracted from vaginal swab eluates using a microbial DNA extraction kit (Tiangen Biotech, Beijing, China) according to the manufacturer’s instructions. DNA concentration and purity were assessed spectrophotometrically. Species-specific quantitative real-time PCR (qPCR) was used to quantify six target microorganisms, including *Lactobacillus crispatus*, *Lactobacillus iners*, *Lactobacillus jensenii*, *Lactobacillus gasseri*, *Gardnerella vaginalis*, and *Candida albicans*. Bacterial primers were selected from previously published qPCR studies on the vaginal microbiota [10,11,12,13] (Supplementary Table S1), and duplicate wells were included for each sample. PCR amplification was performed on a real-time PCR system using SYBR Green chemistry, followed by melting-curve analysis to confirm amplification specificity. No-template negative controls were included in each run to monitor contamination. Absolute quantification was performed using the standard curve method. Standards containing the target amplicons were serially diluted 10-fold to generate standard curves relating cycle threshold values to log10 copy number. A corresponding standard curve was included in each assay batch. The copy number of each target microorganism in the samples was interpolated from the relevant standard curve and back-calculated according to template input volume and nucleic acid extraction/elution volume. Results were expressed as copies per microliter of extracted nucleic acid (copies/μL). Standard copy number was calculated using the following formula: copies/μL = 6.022 × 10^23^ × DNA concentration (g/μL)/[amplicon length (bp) × 660]. For subsequent analyses, copy numbers were log-transformed to approximate a normal distribution.

### 2.4. Treatment and Follow-Up

After completion of baseline assessment, all participants initiated local estriol vaginal cream therapy. Each dose consisted of 0.5 g of cream containing 0.5 mg of estriol, administered once daily for 12 consecutive weeks. Standardized medication instructions were provided to improve adherence. All participants returned for follow-up at week 12 and underwent repeated symptom assessment, gynecological examination, and relevant laboratory testing.

### 2.5. Follow-Up and Outcome Assessment

The primary outcome was clinical symptom improvement at week 12, determined according to the change in MBS score. Improvement in MBS was defined as the baseline MBS score minus the week-12 MBS score, with larger values indicating greater symptom improvement. Consistent with outcome definitions used in previous GSM clinical studies [14], participants with an improvement of at least 2 points in MBS score from baseline to week 12 were classified as responders, whereas those with an improvement of 0–1 point were classified as non-responders.

Secondary outcomes included improvement in VMI and reduction in vaginal pH. Although the classical vaginal maturation index is based on the distribution of parabasal, intermediate, and superficial epithelial cells, in the present study we used the proportion of superficial cells as the primary indicator of epithelial maturation because it directly reflects estrogen-related maturation of the vaginal epithelium and provides a simple, clinically interpretable measure of treatment-related change. An increased proportion of superficial cells was therefore considered to indicate VMI improvement [14].

### 2.6. Statistical Analysis

All statistical analyses were performed using R software (v4.5.3). Continuous variables were first assessed for distributional characteristics. Approximately normally distributed variables are presented as mean ± standard deviation (SD), non-normally distributed variables as median (interquartile range, IQR), and categorical variables as number and percentage [*n* (%)]. Baseline characteristics of the study population were summarized descriptively. Participants were further grouped according to 12-week treatment response status. Between-group comparisons of continuous variables were performed using the independent-samples *t* test or Wilcoxon rank-sum test, as appropriate; categorical variables were compared using Pearson’s χ^2^ test, or Fisher’s exact test when expected cell counts were small. To evaluate changes in the vaginal microbiota and related clinical indicators before and after local estrogen therapy, paired comparisons were performed. For approximately normally distributed continuous variables, paired *t* tests were used, and treatment-related changes were described using means and standard deviations when appropriate. Variables compared between baseline and week 12 included the abundance of six target microorganisms, MBS score, vaginal pH, and VMI.

The primary outcome was 12-week treatment response. To examine associations between baseline target microorganism abundance and treatment response, modified Poisson regression models with robust variance estimation were used to calculate risk ratios (RRs) and 95% confidence intervals (CIs). Univariable and multivariable models were constructed. Multivariable models included clinically relevant covariates. To evaluate the discriminative ability of baseline target microorganism abundance for predicting 12-week treatment response, receiver operating characteristic (ROC) curve analysis was performed, and the area under the curve (AUC) and corresponding 95% CI were calculated. The optimal cutoff was determined using the Youden index, and the corresponding sensitivity, specificity, and 95% CIs were estimated. For indicators with inverse directionality, ROC analyses were aligned so that higher values consistently indicated a higher probability of response, thereby ensuring comparable directionality across indicators.

In addition, linear regression models were used to explore associations between baseline target microorganism abundance and the magnitude of clinical improvement after treatment, with change values in each outcome used as dependent variables. Change was defined as the week-12 value minus the baseline value. Associations between the baseline abundance of the six target microorganisms and changes in MBS, vaginal pH, and VMI were evaluated separately, and regression coefficients, 95% CIs, and *p* values were reported. Given the limited number of preselected target microorganisms and the exploratory nature of the analyses, formal adjustment for multiple comparisons was not applied; therefore, the findings should be interpreted cautiously. All tests were two-sided, and *p* < 0.05 was considered statistically significant.

## 3. Results

A total of 216 postmenopausal women with GSM were included. The mean age was 59.24 ± 5.41 years, the mean body mass index was 23.64 ± 3.15 kg/m^2^, and the median years since menopause was 7 (IQR, 5–12) years. Most participants reported sexual activity within the preceding 3 months (81.5%). At baseline, participants had a substantial symptom burden, with a median MBS score of 3.00 (IQR, 2.00–3.00). In addition, 84.3% of participants had a superficial cell proportion <5%, and the mean vaginal pH was 6.36 ± 0.23, indicating insufficient vaginal epithelial maturation and a markedly hypoestrogenic vaginal microenvironment. Regarding target microorganisms, *L. iners* had the highest abundance (5.20 ± 0.73 log10 copies/μL), whereas *L. jensenii*, *L. gasseri*, *Gardnerella*, and *Candida* showed relatively lower abundances, suggesting considerable heterogeneity in the baseline vaginal microbiota of this population (Table 1).

**Figure 2:**
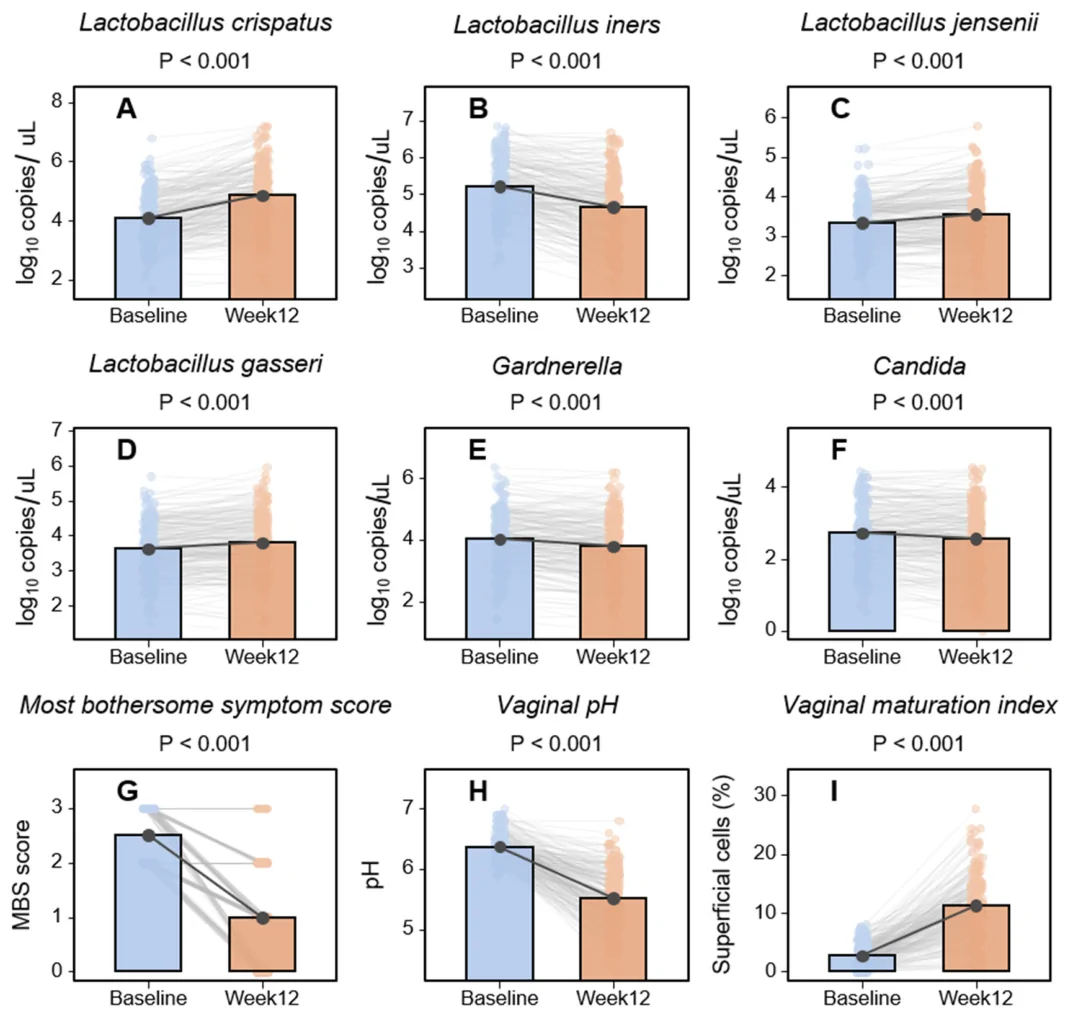
Paired changes in vaginal microbiota, symptoms, and vaginal health indicators from baseline to week 12. *p* values were derived from paired *t* tests. Gray lines indicate paired measurements from the same participant at baseline and week 12, and the bars show the mean values. (**A**) *Lactobacillus crispatus*; (**B**) *Lactobacillus iners*; (**C**) *Lactobacillus jensenii*; (**D**) *Lactobacillus gasseri*; (**E**) *Gardnerella*; (**F**) *Candida*; (**G**) Most bothersome symptom; (**H**) Vaginal pH; (**I**) Vaginal maturation index.

After 12 weeks of treatment, the composition of the vaginal microbiota and related clinical indicators changed significantly from baseline (all *p* < 0.001, paired *t* test; Figure 2). The abundance of *Lactobacillus crispatus*, *L. jensenii*, and *L. gasseri* increased markedly, whereas the abundance of *L. iners*, *Gardnerella*, and *Candida* decreased. In parallel, MBS score and vaginal pH decreased significantly, while VMI increased, indicating reduced symptom burden and improvement in vaginal acidity and epithelial maturation after local estradiol therapy. The corresponding raw data are presented in Supplementary Table S2.

**Figure 3:**
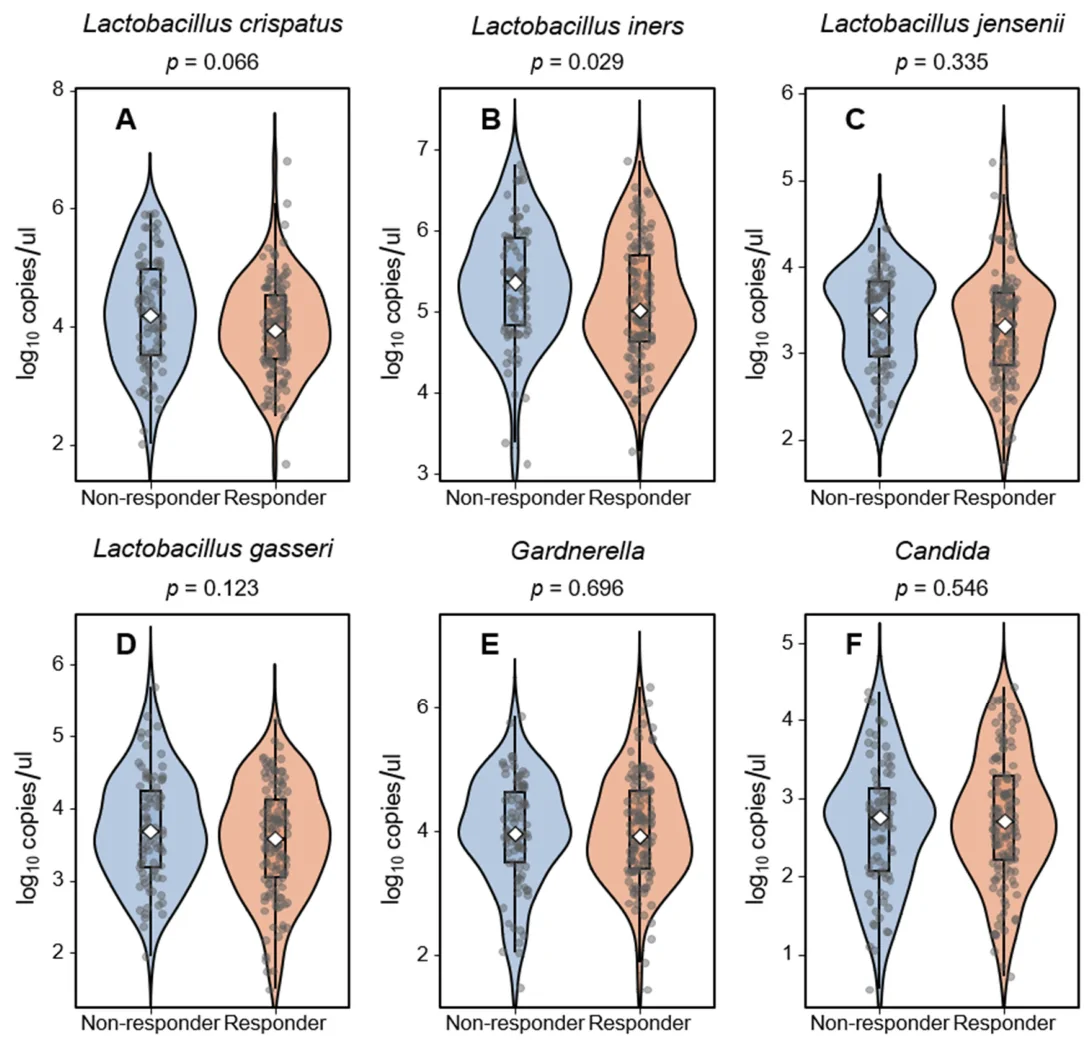
Baseline target microbial abundance according to treatment response. Grey dots represent individual values, and white dots denote the median. (**A**) *Lactobacillus crispatus*; (**B**) *Lactobacillus iners*; (**C**) *Lactobacillus jensenii*; (**D**) *Lactobacillus gasseri*; (**E**) *Gardnerella*; (**F**) *Candida*.

Among the 216 participants, 128 met the predefined treatment response criterion, corresponding to a response rate of 59.3% (95% CI, 52.6–65.6%). The association between baseline target microorganism abundance and treatment response is shown in Figure 3. Compared with non-responders, responders had significantly lower baseline abundance of *L. iners* (*p* = 0.029). In addition, baseline *L. crispatus* abundance tended to be higher in responders, although the difference did not reach statistical significance (*p* = 0.066). No significant differences in baseline abundance of *L. jensenii*, *L. gasseri*, *Gardnerella*, or *Candida* were observed between responders and non-responders (all *p* > 0.05).

After stratification by 12-week treatment response status, responders and non-responders did not differ significantly in age, BMI, years since menopause, sexual activity within the preceding 3 months, smoking status, diabetes, hypertension, history of recurrent vaginitis, baseline MBS score, vaginal pH, or proportion of superficial cells (all *p* > 0.05; Supplementary Table S3).

As shown in Table 2, modified Poisson regression demonstrated a stable inverse association between baseline *L. iners* abundance and treatment response. When analyzed as a continuous variable, *L. iners* abundance was associated with a lower probability of response in the crude model (Model 1: RR, 0.84; 95% CI, 0.72–0.99). This association persisted after adjustment for age, BMI, and years since menopause (Model 2: RR, 0.83; 95% CI, 0.71–0.98), and remained robust after further adjustment for smoking, diabetes, history of recurrent vaginitis, and baseline MBS score (Model 3: RR, 0.82; 95% CI, 0.70–0.95). In tertile-based analyses, compared with the lowest tertile, the highest tertile of *L. iners* abundance was associated with a significantly lower probability of treatment response across all three models, including the fully adjusted model (Model 3: RR, 0.71; 95% CI, 0.54–0.93). In contrast, no clear associations were observed for the other target microorganisms. *L. crispatus* showed a borderline inverse association in the fully adjusted continuous model (Model 3: RR, 0.87; 95% CI, 0.76–1.00), but tertile-based analyses did not show significant differences. *L. jensenii*, *L. gasseri*, *Gardnerella*, and *Candida* showed no statistically significant associations in either continuous or categorical analyses.

**Figure 4:**
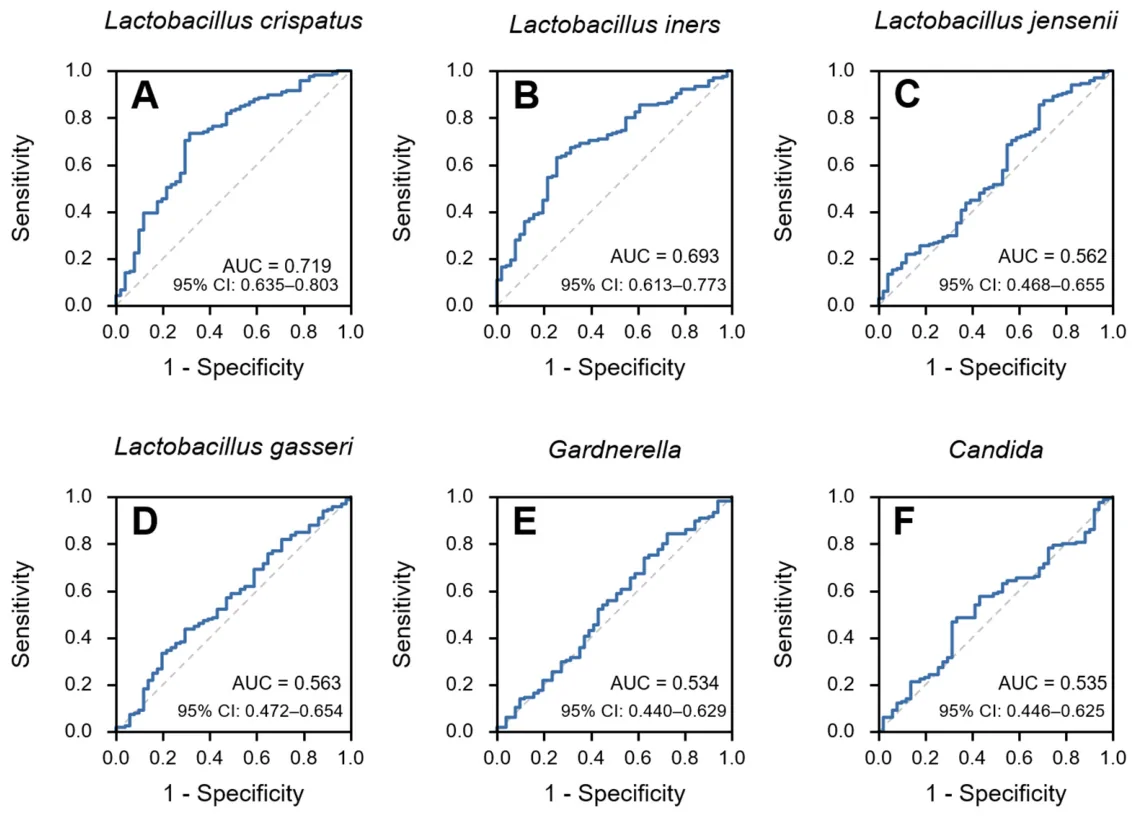
ROC curves for baseline target microbial abundance in predicting treatment response. The blue line represents the ROC curve, and the gray dashed diagonal line indicates the line of no discrimination (AUC = 0.5). (**A**) *Lactobacillus crispatus*; (**B**) *Lactobacillus iners*; (**C**) *Lactobacillus jensenii*; (**D**) *Lactobacillus gasseri*; (**E**) *Gardnerella*; (**F**) *Candida*.

ROC curve analysis (Figure 4) showed that baseline abundance of *L. crispatus* and *L. iners* had moderate discriminative ability for predicting 12-week treatment response. *L. crispatus* had the highest AUC (0.719; 95% CI, 0.635–0.803), followed by *L. iners* (AUC, 0.693; 95% CI, 0.613–0.773). In contrast, the AUCs for *L. jensenii*, *L. gasseri*, *Gardnerella*, and *Candida* were 0.562 (95% CI, 0.468–0.655), 0.563 (95% CI, 0.472–0.654), 0.534 (95% CI, 0.440–0.629), and 0.535 (95% CI, 0.446–0.625), respectively, all close to 0.5, indicating limited predictive utility when used individually.

Overall, baseline target microorganism abundance showed limited correlations with changes in MBS, vaginal pH, and superficial cell proportion after treatment, with low R^2^ values in most models. Baseline *L. crispatus* abundance was modestly associated with the magnitude of pH reduction and increase in superficial cell proportion, and *L. iners* was also associated with change in pH. No clear associations were observed between the remaining microorganisms and these outcome changes, as shown in Supplementary Figures S1–S3.

## 4. Discussion

This study focused on heterogeneity in response to local estrogen therapy among postmenopausal women with GSM. We found that, after 12 weeks of local estradiol vaginal tablet therapy, MBS score, vaginal pH, and VMI improved significantly, accompanied by favorable changes in the vaginal microbiota, including increases in *L. crispatus*, *L. jensenii*, and *L. gasseri* and decreases in *L. iners*, *Gardnerella*, and *Candida*. Using improvement in MBS as the primary outcome, the overall response rate was 59.3%. Baseline *L. iners* abundance was consistently associated with a lower probability of treatment response. Although *L. crispatus* showed relatively good discriminative performance in ROC analysis, its association in regression models was less strong and less stable than that of *L. iners*. Overall, these findings suggest that pretreatment vaginal microbial features, particularly *L. iners* abundance, may represent promising candidate markers for identifying postmenopausal women with GSM who are less likely to benefit from local estrogen therapy, although further external validation is needed before clinical application.

First, this study confirmed significant improvement in GSM-related symptoms, local vaginal physicochemical conditions, and epithelial maturation after local estradiol therapy, consistent with previous evidence that local estrogen can lower vaginal pH, improve vaginal atrophy, and facilitate restoration of the vaginal microenvironment [6]. Existing studies suggest that estrogen promotes vaginal epithelial thickening and glycogen deposition, thereby creating a more favorable environment for *Lactobacillus* colonization and indirectly strengthening lactic acid production and the local acidic barrier [15]. The observed increases in *Lactobacillus*-related taxa and decreases in *Gardnerella* and *Candida* after treatment further support the view that local estrogen therapy may shift the vaginal ecosystem from a hypoestrogenism-related dysbiotic state toward a more stable state. Notably, although most microbial changes occurred in parallel with clinical improvement, correlation analyses suggested that linear associations between individual taxa and changes in MBS, pH, or superficial cells were generally weak. This implies that treatment response in GSM is unlikely to be determined by a single microorganism and more likely reflects interactions among the vaginal epithelium, hormonal milieu, and microbiota.

The most important finding of this study was that higher baseline *L. iners* abundance was consistently associated with a lower probability of treatment response. Unlike *L. crispatus* and other *Lactobacillus* species typically regarded as dominant protective taxa, *L. iners* has a smaller genome, more limited metabolic capacity, and can be detected across both healthy and dysbiotic vaginal states [8,16]. Therefore, high *L. iners* abundance does not necessarily indicate a truly stable or protective vaginal microenvironment; rather, it may represent a transitional ecological state that is fragile but not yet overtly dysbiotic. However, the biological role of *L. iners* is likely context-dependent and may vary according to the host environment and the broader microbial community structure, such that it may be observed in both transitional and relatively stable microbial states. Previous studies have also suggested that *L. iners*-dominated communities are less stable than *L. crispatus*-dominated communities and more prone to transition toward abnormal vaginal states [15,17]. In this context, our findings may be interpreted as follows: patients with higher pretreatment *L. iners* abundance may retain some *Lactobacillus*-related features but have insufficient ecological stability, resulting in a poorer response to local estrogen therapy. Thus, in the present study, *L. iners* is better understood as a marker of microbial fragility or ecological transition rather than a simply protective taxon.

By contrast, *L. crispatus* showed relatively good discriminative ability in ROC analysis, but its associations in between-group comparisons and multivariable regression were less stable than those of *L. iners*. This observation is informative. *L. crispatus* has long been associated with lower vaginal pH, stable *Lactobacillus*-dominant communities, and reduced infection risk [18,19], and its predictive signal for treatment response is therefore biologically plausible. However, *L. crispatus* may primarily reflect an overall healthy or stable ecological background, whereas *L. iners* may more sensitively capture transition from stability toward fragility. Therefore, for predicting short-term response to local estrogen therapy, *L. iners* may be closer to the ecological signal underlying response heterogeneity than *L. crispatus*.

In this study, *L. jensenii*, *L. gasseri*, *Gardnerella*, and *Candida* did not show stable independent predictive associations. This finding suggests that not all target microorganisms equally reflect differences in treatment response among postmenopausal women with GSM. One possible explanation is that these taxa may mainly reflect broader ecological background rather than directly determine treatment benefit. Another explanation is that this study quantified a limited panel of target microorganisms and could not fully capture the entire vaginal microbial network. Future studies incorporating higher-resolution microbial sequencing and functional omics approaches may further clarify the complex relationship between the postmenopausal vaginal ecosystem and response to hormone therapy.

This study has potential clinical implications. First, pretreatment assessment of the vaginal microbiota, especially *L. iners* abundance, may have potential to help identify patients who are less likely to benefit from local estrogen therapy. For such patients, closer monitoring of treatment response or individualized strategies incorporating microbiota-targeted interventions may be considered in the future. Second, this study integrated symptom scores, vaginal pH, VMI, and absolute quantification of target microorganisms, thereby providing a more comprehensive assessment of the clinical and biological dimensions of response to local estrogen therapy and offering preliminary evidence for precision stratification in the management of postmenopausal GSM. If validated in future studies, pretreatment assessment of vaginal microbial features, particularly *L. iners* abundance, may have potential clinical utility in identifying women at increased risk of suboptimal response to local estrogen therapy. At present, this information should not be used to replace standard treatment selection, but it may eventually support more individualized follow-up strategies, such as earlier reassessment of symptom improvement and vaginal health indicators in patients with higher-risk microbial profiles. In addition, such patients may represent potential candidates for future adjunctive interventions targeting the vaginal microbiota, although these approaches remain investigational. Future studies may further investigate whether adjunctive vaginal or oral lactobacillus-based interventions, in combination with local estrogen therapy, can improve treatment response in women with higher-risk microbial profiles.

Several limitations should be acknowledged. First, this was a single-center study with a relatively concentrated source population; therefore, the generalizability of the findings requires validation in larger and multicenter cohorts. Second, the follow-up period was 12 weeks, and the study primarily reflected short-term treatment response; longer-term clinical benefit and microbial stability could not be evaluated. Third, only a limited panel of preselected target microorganisms was measured by qPCR, and the overall community structure of the postmenopausal vaginal microbiota was not comprehensively characterized. However, the present study was designed to evaluate a focused and clinically feasible microbial assessment strategy rather than to perform comprehensive microbiome profiling. The selected targets were determined a priori based on existing evidence regarding postmenopausal vaginal ecology and GSM-related microbial alterations. broader sequencing-based studies will be necessary to validate and extend our findings at the community level. Fourth, because this was an observational analysis, the stable association between *L. iners* and poorer treatment response should not be interpreted as evidence of causality. Therefore, the observed post-treatment improvements in clinical and microbial indicators cannot be interpreted as exclusively attributable to local estrogen therapy, and the potential influence of non-specific or time-related factors cannot be fully excluded. Future controlled studies are needed to validate these findings. Fifth, although a history of recurrent vaginitis may have clinical relevance to baseline vaginal ecology and treatment-related microbial dynamics, this factor was not analyzed as a prespecified stratification variable in the present study. Future studies should further evaluate whether microbial profiles and response to local estrogen therapy differ according to recurrent vaginitis history. Sixth, GSM encompasses not only vaginal symptoms but also vulvar and lower urinary tract symptoms. The present study primarily evaluated vaginal symptoms and vaginal biological indicators, the potential relevance of baseline vaginal microbial features should currently be interpreted as reflecting mainly the vaginal component of GSM. In addition, because multiple microorganisms were evaluated across several statistical analyses, the possibility of type I error cannot be fully excluded. Although the targets were preselected based on prior biological relevance, the present findings should be interpreted cautiously and require external validation.

## 5. Conclusions

In conclusion, postmenopausal women with GSM experienced significant improvement in symptoms, vaginal environment, and epithelial maturation after local estradiol therapy. Higher baseline abundance of *L. iners* was independently associated with a lower probability of treatment response. These findings suggest that *L. iners* may represent a promising candidate microbial marker associated with response to local estrogen therapy. However, its predictive value remains preliminary, and external validation is required before clinical implementation in individualized treatment or risk stratification of women with GSM.

## Figures and Tables

**Table 1:** Baseline characteristics of the study population.

Variable	*N* = 216
Age (years)	59.24 ± 5.41
BMI (kg/m^2^)	23.64 ± 3.15
Years since menopause, median (IQR)	7 (5, 12)
Sexual activity within the past 3 months	176 (81.5%)
Smoking	8 (3.7%)
Diabetes	23 (10.6%)
Hypertension	45 (20.8%)
History of recurrent vaginitis	65 (30.1%)
Baseline MBS score	3.00 (2.00, 3.00)
Baseline superficial cells < 5%	182 (84.3%)
Baseline vaginal pH	6.36 ± 0.23
*L. crispatus* log10 abundance (copies/μL)	4.08 ± 0.85
*L. iners* log10 abundance (copies/μL)	5.20 ± 0.73
*L. jensenii* log10 abundance (copies/μL)	3.34 ± 0.61
*L. gasseri* log10 abundance (copies/μL)	3.62 ± 0.76
*Gardnerella* log10 abundance (copies/μL)	4.01 ± 0.87
*Candida* log10 abundance (copies/μL)	2.71 ± 0.81

**Table 2:** Associations of baseline target microbial abundance with treatment response using modified Poisson regression.

Microbe	Term	Model 1	Model 2	Model 3
*L. crispatus*	Continuous	0.88 (0.77, 1.01)	0.88 (0.77, 1.01)	0.87 (0.76, 1.00)
	T1	Reference	Reference	Reference
	T2	1.00 (0.78, 1.28)	1.00 (0.78, 1.28)	0.98 (0.76, 1.26)
	T3	0.78 (0.59, 1.04)	0.78 (0.58, 1.05)	0.78 (0.58, 1.04)
*L. iners*	Continuous	0.84 (0.72, 0.99)	0.83 (0.71, 0.98)	0.82 (0.70, 0.95)
	T1	Reference	Reference	Reference
	T2	0.80 (0.62, 1.03)	0.78 (0.60, 1.02)	0.78 (0.60, 1.01)
	T3	0.76 (0.58, 0.99)	0.74 (0.56, 0.97)	0.71 (0.54, 0.93)
*L. jensenii*	Continuous	0.92 (0.76, 1.10)	0.91 (0.76, 1.09)	0.92 (0.76, 1.11)
	T1	Reference	Reference	Reference
	T2	1.09 (0.85, 1.40)	1.10 (0.86, 1.41)	1.10 (0.86, 1.42)
	T3	0.82 (0.61, 1.10)	0.82 (0.61, 1.10)	0.83 (0.62, 1.12)
*L. gasseri*	Continuous	0.89 (0.78, 1.02)	0.90 (0.78, 1.03)	0.89 (0.77, 1.02)
	T1	Reference	Reference	Reference
	T2	1.00 (0.78, 1.29)	1.00 (0.78, 1.29)	1.01 (0.78, 1.30)
	T3	0.84 (0.64, 1.12)	0.84 (0.64, 1.12)	0.83 (0.63, 1.10)
*Gardnerella*	Continuous	1.03 (0.90, 1.17)	1.02 (0.90, 1.16)	1.01 (0.89, 1.16)
	T1	Reference	Reference	Reference
	T2	0.87 (0.67, 1.13)	0.87 (0.67, 1.14)	0.84 (0.65, 1.10)
	T3	0.85 (0.65, 1.11)	0.84 (0.65, 1.10)	0.82 (0.62, 1.08)
*Candida*	Continuous	1.04 (0.91, 1.20)	1.04 (0.91, 1.20)	1.04 (0.90, 1.19)
	T1	Reference	Reference	Reference
	T2	0.95 (0.72, 1.27)	0.95 (0.71, 1.27)	0.94 (0.71, 1.26)
	T3	1.10 (0.84, 1.42)	1.09 (0.84, 1.42)	1.08 (0.83, 1.40)

Data are presented as risk ratio (RR) with 95% confidence interval and *p* value. Model 1 was crude. Model 2 was adjusted for age, BMI, and years since menopause. Model 3 was additionally adjusted for smoking, diabetes, history of recurrent vaginitis, and baseline most bothersome symptom score.

## Data Availability

The original contributions presented in this study are included in the article/Supplementary Materials. Further inquiries can be directed to the corresponding author.

## Supplementary Materials

The following supporting information can be downloaded at: https://www.mdpi.com/article/10.3390/microorganisms14081760/s1, Table S1: Species-specific qPCR primers for six bacterial targets; Table S2: Mean ± SD of vaginal microbiota and clinical indicators at baseline and week 12, with paired *t*-test results; Table S3: Baseline characteristics according to treatment response status; Figure S1: Association between baseline vaginal microbiota abundance and changes in MBS; Figure S2: Association between baseline vaginal microbiota abundance and changes in vaginal pH; Figure S3: Association between baseline vaginal microbiota abundance and changes in superficial cells (%).

## Abbreviations

The following abbreviations are used in this manuscript:

AUC	area under the curve
BMI	body mass index
CI	confidence interval
GSM	genitourinary syndrome of menopause
IQR	interquartile range
ISSWSH	International Society for the Study of Women’s Sexual Health
MBS	most bothersome symptom
NAMS	North American Menopause Society
PCR	polymerase chain reaction
ROC	receiver operating characteristic
RR	risk ratio
SD	standard deviation
VMI	vaginal maturation index
