# Supplementary material for: Vaginal Lactobacillus iners Abundance Predicts Response to Local Estrogen Therapy in Postmenopausal Women with Genitourinary Syndrome of Menopause
Source: Microorganisms. 2026 Aug 10;14(8):1760. doi: 10.3390/microorganisms14081760 (PMC13515633; doi:10.3390/microorganisms14081760)
Supplement: Supplementary file 1 [file microorganisms-14-01760-s001.zip › microorganisms-4448827-supplementary.pdf]

Table S1. Species-specific qPCR primers for six bacterial targets

Target microorganism	Forward primer (5′–3′)	Reverse primer (5′–3′)	Target gene	Primer source	qPCR cycling conditions
Lactobacillus crispatus	AGCGAGCGGAACTAACAGATTTAC	AGCTGATCATGCGATCTGCTT	16S rRNA	Byun et al.	95°C 15 min; 40 cycles of 95°C 15 s, 60°C 60 s, 72°C 20 s
Lactobacillus iners	GTCTGCCTTGAAGATCGG	ACAGTTGATAGGCATCATC	16S rRNA	De Backer et al.	95°C 15 min; 35 cycles of 95°C 15 s, 60°C 55 s, 65°C 60 s
Lactobacillus jensenii	AAGTCGAGCGAGCTTGCCTATAGA	CTTCTTTCATGCGAAAGTAGC	16S rRNA	Tamrakar et al.	95°C 15 min; 40 cycles of 95°C 15 s, 60°C 55 s, 72°C 60 s
Lactobacillus gasseri	AGCGAGCTTGCCTAGATGAATTTG	TCTTTTAAACTCTAGACATGCGTC	16S rRNA	Tamrakar et al.	95°C 15 min; 40 cycles of 95°C 15 s, 57°C 60 s, 65°C 60 s
Gardnerella vaginalis	TTACTGGTGTATCACTGTAAGG	CCGTCACAGGCTGAACAGT	16S rRNA	Zariffard et al.	95°C 15 min; 50 cycles of 95°C 45 s, 55°C 45 s, 72°C 45 s

Reference:

【1】De Backer E, Verhelst R, Verstraelen H, Claeys G, Verschraegen G, Temmerman M, et al. Quantitative determination by real-time PCR of four vaginal Lactobacillus species, Gardnerella vaginalis and Atopobium vaginae indicates an inverse relationship between L. gasseri and L. iners. BMC Microbiol. 2007;7:115. doi:10.1186/1471-2180-7-115.

【2】Byun R, Nadkarni MA, Chhour KL, Martin FE, Jacques NA, Hunter N. Quantitative analysis of diverse Lactobacillus species present in advanced dental caries. J Clin Microbiol. 2004;42(7):3128-3136. doi:10.1128/JCM.42.7.3128-3136.2004.

【3】Tamrakar R, Yamada T, Furuta I, Cho K, Morikawa M, Yamada H, et al. Association between Lactobacillus species and bacterial vaginosis-related bacteria, and bacterial vaginosis scores in pregnant Japanese women. BMC Infect Dis. 2007;7:128. doi:10.1186/1471-2334-7-128.

【4】Zariffard MR, Saifuddin M, Sha BE, Spear GT. Detection of bacterial vaginosis-related organisms by real-time PCR for Lactobacilli, Gardnerella vaginalis and Mycoplasma hominis. FEMS Immunol Med Microbiol. 2002;34(4):277-281. doi:10.1111/j.1574-695X.2002.tb00636.x.

Table S2. Mean ± SD of vaginal microbiota and clinical indicators at baseline and week 12, with paired t-test results.

Variable	N	Baseline	Week 12	Mean difference (Week 12 - Baseline)	t	P value
Lactobacillus crispatus, log10 copies/ul	216	4.08 ± 0.85	4.85 ± 1.00	0.77 ± 0.67	16.94	<0.001
Lactobacillus iners, log10 copies/ ul	216	5.20 ± 0.73	4.65 ± 0.91	-0.56 ± 0.53	-15.35	<0.001
Lactobacillus jensenii, log10 copies/ ul	216	3.34 ± 0.61	3.54 ± 0.74	0.21 ± 0.34	8.96	<0.001
Lactobacillus gasseri, log10 copies/ ul	216	3.62 ± 0.76	3.79 ± 0.85	0.16 ± 0.36	6.74	<0.001
Gardnerella, log10 copies/ ul	216	4.01 ± 0.87	3.78 ± 0.97	-0.23 ± 0.47	-7.23	<0.001
Candida, log10 copies/ ul	216	2.71 ± 0.81	2.56 ± 0.92	-0.15 ± 0.42	-5.32	<0.001
Most bothersome symptom score	216	2.50 ± 0.50	0.99 ± 0.85	-1.52 ± 0.88	-25.40	<0.001
Vaginal pH	216	6.36 ± 0.23	5.51 ± 0.48	-0.85 ± 0.46	-27.01	<0.001
Vaginal maturation index, superficial cells (%)	216	2.67 ± 2.09	11.20 ± 5.23	8.53 ± 5.02	24.97	<0.001

Abbreviation: SD, standard deviation.

Table S3. Baseline characteristics according to treatment response status.

Variable	Overall	Responder (n=128)	Non-responder (n=88)	P value
Age (years)	59.24 ± 5.41	59.07 ± 5.31	59.47 ± 5.57	0.599
BMI (kg/m²)	23.64 ± 3.15	23.59 ± 3.26	23.70 ± 3.01	0.807
Years since menopause	8.96 ± 4.94	9.25 ± 4.63	8.53 ± 5.37	0.310
Sexual activity within the past 3 months	176 (81.5%)	109 (85.2%)	67 (76.1%)	0.094
Smoking	8 (3.7%)	4 (3.1%)	4 (4.5%)	0.718
Diabetes	23 (10.6%)	12 (9.4%)	11 (12.5%)	0.464
Hypertension	45 (20.8%)	25 (19.5%)	20 (22.7%)	0.570
History of recurrent vaginitis	65 (30.1%)	41 (32.0%)	24 (27.3%)	0.454
Baseline most bothersome symptom score	2.50 ± 0.50	2.53 ± 0.50	2.47 ± 0.50	0.348
Baseline superficial cells <5%	182 (84.3%)	107 (83.6%)	75 (85.2%)	0.746
Baseline vaginal pH	6.36 ± 0.23	6.39 ± 0.22	6.33 ± 0.24	0.084
Baseline superficial cells (%)	2.67 ± 2.09	2.65 ± 1.99	2.69 ± 2.23	0.883

P values were derived from Student's t-test for continuous variables and chi-square test or Fisher's exact test for categorical variables, as appropriate.

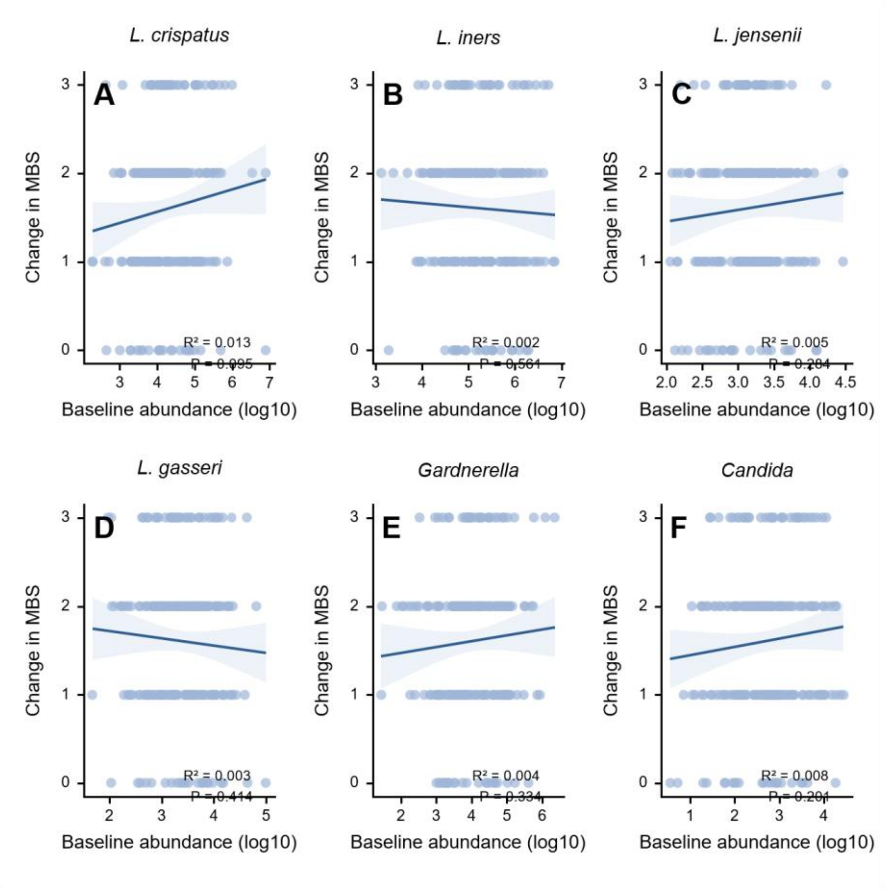

Figure S1. Association between baseline vaginal microbiota abundance and changes in MBS.

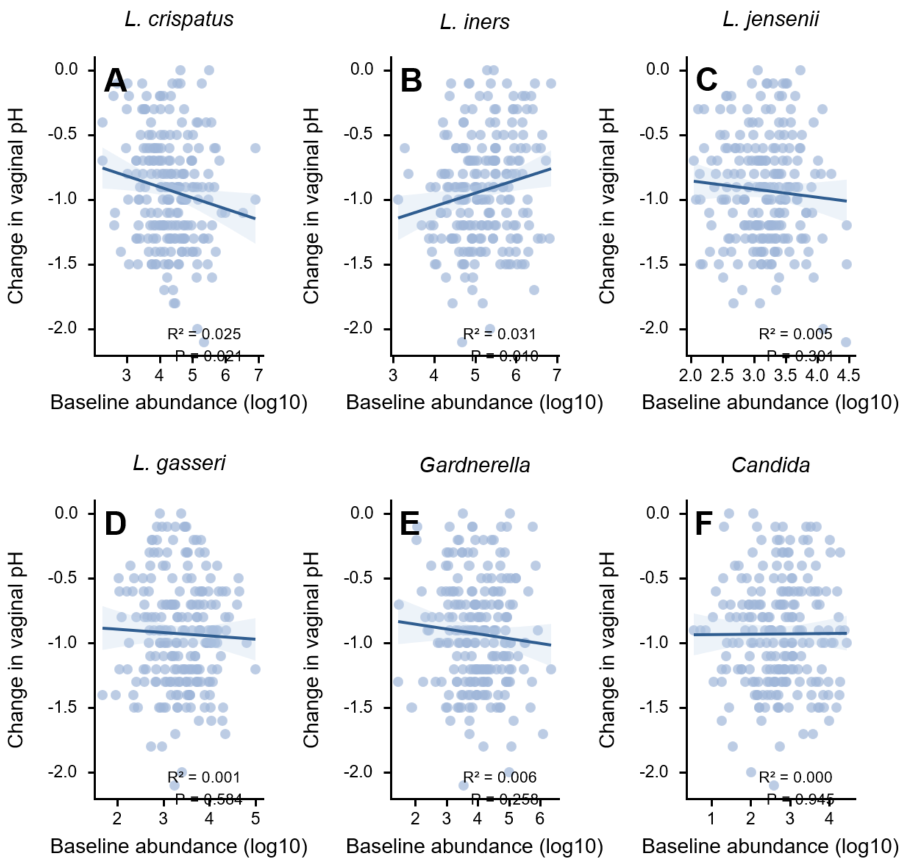

Figure S2. Association between baseline vaginal microbiota abundance and changes in vaginal pH.

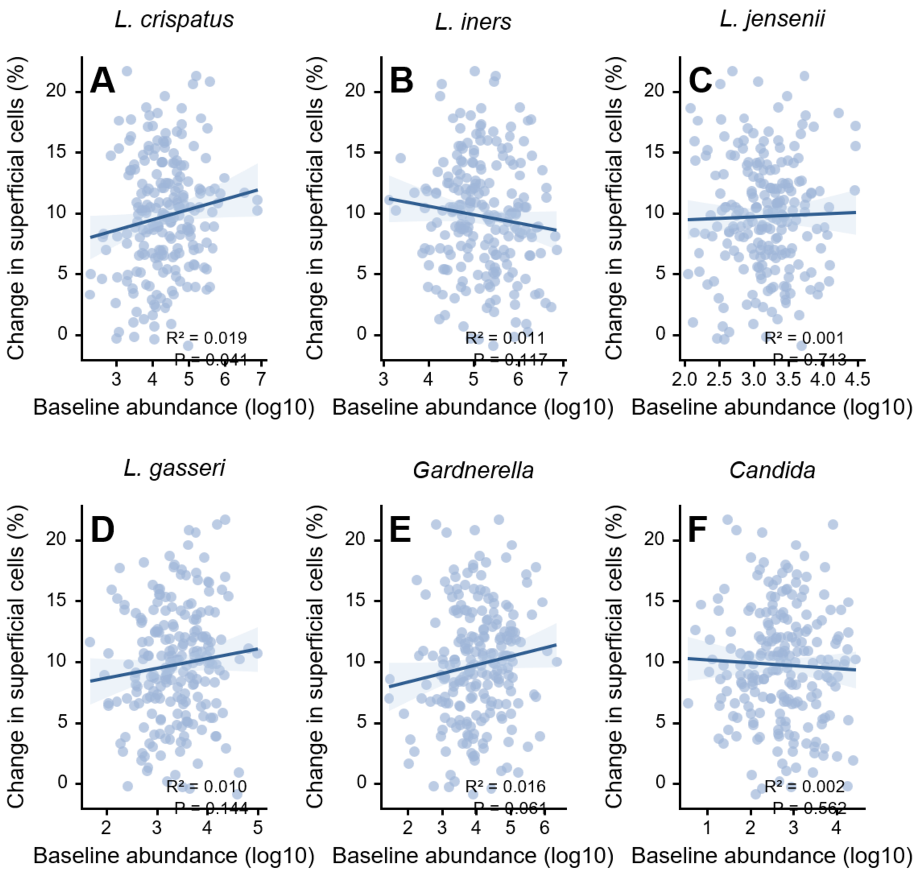

Figure S3. Association between baseline vaginal microbiota abundance and changes in superficial cells (%).
